# Vaccine Candidates against Nontypeable *Haemophilus* *influenzae:* a Review

**DOI:** 10.18869/acadpub.ibj.21.2.69

**Published:** 2017-03

**Authors:** Ava Behrouzi, Farzam Vaziri, Fatemeh Rahimi-Jamnani, Parviz Afrough, Mohammad Rahbar, Fereshteh Satarian, Seyed Davar Siadat

**Affiliations:** 1 Department of Mycobacteriology and Pulmonary Research, Pasteur Institute of Iran, Tehran, Iran; 2 Microbiology Research Center, Pasteur Institute of Iran, Tehran, Iran; 3 Department of Microbiology, Reference Health Laboratories Research Center, Ministry of Health and Medical Education, Tehran, Iran

**Keywords:** Vaccines, Chronic obstructive pulmonary disease, *Haemophilus influenzae*

## Abstract

Nonencapsulated, nontypeable *Hemophilus* *influenzae* (NTHi) remains an important cause of acute otitis and respiratory diseases in children and adults. NTHi bacteria are one of the major causes of respiratory tract infections, including acute otitis media, cystic fibrosis, and community-acquired pneumonia among children, especially in developing countries. The bacteria can also cause chronic diseases such as chronic bronchitis and chronic obstructive pulmonary disease in the lower respiratory tract of adults. Such bacteria express several outer membrane proteins, some of which have been studied as candidates for vaccine development. Due to the lack of effective vaccines as well as the spread and prevalence of NTHi worldwide, there is an urgent need to design and develop effective vaccine candidates against these strains.

## INTRODUCTION

*H* *aemophilus influenzae* was first isolated from sputum samples of patients suffering from influenza during influenza pandemics in 1892. However, the bacterium was mistakenly considered as causative agent of influenza disease until 1933, when the viral etiology of influenza became known[1]. In 1931, encapsulated and nonencapsulated *H. influenzae* strains were introduced and six various serotypes (a, b, c, d, e, and f) were found for encapsulated *H. influenzae*. Among them, *H. influenzae* type b (Hib) is known to be a major virulent pathogen[2-4]. Hib conjugate vaccines are able to eradicate the invasive Hib disease in children throughout the world. Additionally, such conjugate vaccines can induce protective humoral immune responses and decline circulating Hib strains among population through decreased nasopharyngeal carriage of Hib. Taken together, it can be deduced that such conjugate vaccines significantly affect the incidence of *H. influenzae* disease and the subsequent colonization in the respiratory tract[5-9].

### Microbiology

*H. influenzae* is a small, nonmotile, Gram-negative coccobacillus that requires special growth factors, such as X (hemin) and V (*nicotinamide adenine dinucleotide*). The bacterium is generally an anaerobe but can grow as facultative anaerobic organisms. *H. influenzae* can be found, as a part of the normal microflora, in the upper respiratory tractespecially oropharynx and nasopharynx, of about 90% of adults. Therefore, *H. influenzae* may act as opportunistic pathogens, particularly in patients suffering from viral infections or compromised immune systems[10].

### Diseases caused by *haemophilus*

Hib is responsible for several infections such as bactremia, epiglottitis, otitis media, acute pharyngitis bronchitis pneumonia, acute bacterial meningitis, endocarditis, and conjunctivitis in infants and small children. The emergence of Hib protein-polysaccharide conjugate vaccines, in the late 1980s, led to the control and elimination of Hib-related diseases and Hib carriage rates in several industrialized countries[11]. Encapsulated *H. influenzae* strains, except for serotype b, have long been known to be nonvirulent strains associated with rare diseases. Nevertheless, the replacement of Hib with other capsule types has been proposed as a candidate for vaccines against invasive diseases caused by *H. influenzae* serotypes a, e, and f. Nonencapsulated, nontypeable *H. influenzae* (NTHi) bacteria are one of the major causes of respiratory tract infections, including acute otitis media (AOM), cystic fibrosis, and community-acquired pneumonia among children, especially in developing countries The bacteria are also known to cause chronic bronchitis and chronic obstructive pulmonary disease (COPD) in the adult lower respiratory tract[12].

Nontypeable *H. influenzae*, as the main etiology of otitis media, can be established and developed in the lower respiratory tract of adults with COPD. It is important to note that COPD is the fourth leading cause of mortality around the world, which results in disease exacerbation[13,14].

NTHi bacteria have been indicated to play a role in the formation of biofilms in the respiratory tract of adults with COPD, similar to that observed with *Pseudomonas aeruginosa* in patients with cystic fibrosis[15]. Biofilms are a unique community of bacterial cells surrounded by their own polymer matrix that attaches to solid surfaces. Approximately 99.9% of the bacteria naturally exhibit a biofilm on different surfaces, which, unlike planktonic bacteria, have the ability to develop resistance to antibiotics and immune clearance mechanisms. Therefore, it is necessary to clarify the importance of biofilm formation in bacterial pathogens for the development of appropriate approaches to control infections caused by bacteria in biofilms such as otitis media with effusion. NTHis, *Streptococcus pneumoniae* and *Moxarella catarrhalis*, are believed to be major causes of adult pneumonia. However, the virulence of NTHi is typically less than that of *S*. *pneumonia*; NTHi bacteria are often associated with underlying lung diseases[14,16,17].

### Virulence factors of NTHi

#### Fimbrial adhesion

Fimbriae, as a colonization factor, are appendages of *H. influenzae* that facilitate bacterial adherence to human cells. Fimbriae play an important role in the first step of infections through bacterial adherence to human mucosal epithelial cells.

#### Lipooligosaccharide (LOS)

LOSs are cell wall-associated glycolipids composed of a lipid A linked to a series of oligosaccharide components (with various molecular sizes) through 2-keto-3-deoxyoctulosonic acid. The LOS is considered as a surface adhesion that helps *H. influenzae* colonization in the airway[18].

#### IgA proteases

IgA proteases are endopeptidases (types 1 and 2) secreted by NTHi to cleave and neutralize immune-globulin A1. Studies have demonstrated that almost all of the NTHi strains can synthesize at least one of these IgA proteases[19]. These enzymes are able to inactivate human IgA1 present in the nasopharynx, leading to the inactivation of more than 90% of IgA1s[20]. More importantly, recent studies have demonstrated that almost all NTHi strains are able to secrete the IgA protease[21].

#### Opacity-associated protein A (OapA)

All *H. influenzae* strains have an OapA with a low molecular weight (47 kDa). OapA plays important roles in pharyngeal colonization and bacterial adherence to epithelial cells in cell culture[22].

### Epidemiology

Nontypeable *H. influenzae* strains are considered to be one of the most common commensal organisms in the human nasopharynx. Approximately 20% of infants within the first year of their life are colonized with nontypeable *H. influenzae* strains, followed by high-level colonization during adulthood. Unlike adults who typically carry only one type, children are often colonized with multiple strains simultaneously[23].

Based on statistics, NTHi strains are responsible for 20-30% of all episodes of AOM and possibly a higher percentage of recurrent episodes. In addition, NTHi strains account for more than 40% of otitis media with effusion cases (chronic otitis media), and about one-third of acute or chronic sinusitis is also caused by NTHi strains. NTHi can be considered as the cause of chronic bronchitis, pulmonary exacerbations, and community-acquired pneumonia, especially among children living in developing countries, patients with underlying chronic lung disease, as well as the elderly individuals. In some cases, *H. influenzae* has been introduced as the agent responsible for systemic diseases such as meningitis, septicemia, and septic arthritis[24,25].

Nasopharyngeal NTHi colonization leads to the risk of respiratory tract diseases[26]. Smoking results in goblet cell hyperplasia, mucus hypersecretion, and decreased respiratory epithelial cell ciliary function, presumably elevating the localization of NTHi-mediated respiratory tract diseases. Underlying anatomic abnormalities or compromised immunities make patients prone to systemic diseases caused by NTHi[27]. Although NTHi-related diseases can be successfully treated with the commonly used β-lactam antibiotics such as ampicillin or amoxicillin, resistance among the bacteria is rising increasingly through different mechanisms such as β-lactamase[26] or through modified penicillin-binding proteins with low affinity for β-lactams. In addition, various studies have reported the emergence of resistance to trimethoprim-sulfamethoxazole, clarithromycin, and azithromycin. One of the important dimensions of this phenomenon is economic issues, including the cost of physician visits and medications, which is estimated around $1 billion per year in the United States[28,29].

### Vaccines

There are a variety of effective vaccines widely used against Hib strains; however, none of which provide enough protection to children. It is well-documented[30, 31] that protein carriers of polyribosylribitol phosphate (PRP)-conjugated vaccines have the potential ability to induce antibody responses in infants at the peak age incidence of Hib infection.

Antibodies generated against PRP, the polysaccharide capsule of *Hib*, provide protection against the Hib disease. However, the purified PRP used in the first generation of Hib vaccines failed to induce adequate immune responses in children younger than 18 months, who are too susceptible to the Hib disease. Carrier protein PRP-conjugates leads to improved immunogenicity of the vaccine and generation of a protective response to Hib diseases in young infants. In spite of their sustained immune-genicity and high efficacy, Hib conjugate vaccines failed to provide protection against Hib infections in a small number of infants. The children vaccinated with Hib seem to have a defect in immunological priming with lower-affinity antibodies to Hib capsular polysaccharide. some investigations[32,33] have been conducted to find novel vaccines based on some of the surface-exposed *H. influenzae* proteins such as pili and outer membrane proteins (OMPs). Such vaccines reflect high protective efficacies against Hib and NTHi infections. Nonetheless, there is a need for further studies to characterize bacterial structures for discovery of protective vaccine antigens. This goal may not be easily achieved in practice for such bacteria due to their extensive sequence and antigenic variations among gene products interacting with the immune system such as OMPs, adhesins, lipopolysaccharides (LPSs) and secreted virulence factors[32-39].

### Vaccination strategies for NTHi infections

Despite the widespread availability of antibiotics, NTHi infections are considered as major causes of morbidity and mortality, which emphasizes the importance of development of protective vaccine candidates against NTHi infections. However, it is difficult to develop an appropriate vaccine because NTHi strains, as commensal bacteria, are predominantly present on mucosal surfaces, particularly in the airways as parts of natural flora. These organisms are genetically very diverse; therefore, there are no genetic markers to differentiate NTHi strains by the existing typing systems.

There are antibodies against OMPs and LPSs in human serum. Therefore, most of the studies on human immunity against NTHi infections have focused on identification of those OMPs with immunogenic and antigenic properties that are essential for colonization, invasion, and survival within the human host[40]. These low-molecular-weight proteins of *H. influenzae* are classified into major OMPs, including P1, P2, and P4-P6, and minor OMPs, including the transferrin binding protein 1, 2 (Tbp1/Tbp2) and protein D. The use of OMP P2, as a vaccine candidate, has been limited due to its highly heterogeneity and subsequent antigenic drift during persistent infections in patients with chronic bronchitis[41,42].

### Outer membrane proteins: vaccine candidates

#### P1

P1 is a heat-modifiable protein with a molecular mass of 35 kDa at room temperature and 46–50 kDa after boiling. Even though exhibiting high antigenic variations in its surface-exposed epitopes, P1 is highly immunogenic, which can induce protective antibodies against NTHi-induced otitis media in chinchillas. Relatively protective efficiency of antibodies generated by P1 has been studied in the infant rat bacteremic model[43].

#### P2

P2 is the most abundant OMP with a molecular mass ranging from 36 to 42 kDa. The protein can be found in all strains as a trimeric porin structure, allowing molecules up to 1400 Da to diffuse across the membrane. Different NTHi strains are highly variable in P2 amino acid sequences. There are 16 relatively conserved transmembrane regions and 8 heterogeneous surface-exposed loops in P2[44,45].

#### P4

OMP P4 is a highly-conserved cationic lipoprotein with molecular mass of 28 to 30 kDa, designated as lipoprotein *e*, in all strains of *H. influenzae*[20]. The presence of hemin-binding motifs within P4 and the construction of *H. influenzae hel* mutants incapable of aerobic growth indicate the vital role of P4 in heme acquisition[20]. In spite of the fact that lipoprotein *e* plays a role in transporting exogenous heme into the cell, the biochemistry of heme binding and transport remains to be elucidated[46,47].

#### P6

P6, with a molecular mass of 16 kDa, is one of the significant outer membrane lipoproteins that plays a strong immunoregulatory role similar to other lipoproteins containing an N-terminal tri-palmitoyl cysteine (Cys-Pam3) motif. P6 significantly induces the secretion of IL-8 and TNF-a in macrophages. The protein, as a common determinant among all typeable and nontypeable *H. influenzae* strains, has one epitope that is highly conserved among nontypeable strains. P6 has been suggested as an important antigen in human immunity to *H. influenzae*. Antibodies produced against P6 protein with peptidoglycan from a type b strain indicated a protective role for infant rats in a model of *H. influenzae* infection. A monoclonal antibody of (7F3) raised against P6 has the ability to disrupt bactericidal activity of human serum against NTHi. Bactericidal activity of serum for NTHi was reduced by depleting normal human serum of antibody to P6 by the affinity chromatography[48-51].

#### Tbp1 and Tbp2

*In vitro* experiments have shown that *H. influenzae* is able to absorb iron from human transferrin through two transferring-binding proteins, Tbp1 and Tbp2, expressed on bacterial cell surfaces. These proteins are produced during infection probably to acquire iron *in vivo*. The lack of these proteins severely impairs the bacterial growth. Various studies have demonstrated[52] the presence of antibodies against both Tbp1 and Tbp2 in convalescent-phase sera. Genes-encoding Tbp1 (tbpA) and Tbp2 (tbpB) in *H. influenzae* have recently been cloned and sequenced. Accordingly, Tbp1 has been demonstrated to play a vital role in the growth of isogenic mutants in human transferrin, while a mutant with no Tbp2 showed severely limited growth on transferrin-bound iron[53,54].

#### D protein

Protein D (PD) is originally a surface-exposed OMP of *H. influenzae* with the ability to bind to the human IgD myeloma protein 4490[55]. Cloning and characterization of the *hpd* gene from *H. influenzae* indicated that PD, as an *hpd* gene product, binds to the 125I-labeled IgD myeloma protein. The interaction between *H. influenzae* and the immune system is occurred by the binding of IgD to its surface[56]; this interaction is shared with *Moraxella* *(Branhamella) catarrhalis* with unknown reasons. All 127 *H. influenzae* strains, including encapsulated serotypes a–f and NTHi, have this antigenically conserved 42-kDa protein[57], making it an attractive vaccine candidate. A 1092 bp open reading frame of *hpd* encodes a 364-amino acid protein. The nucleotide and deduced amino acid levels have 97% identical sequences, and the substitutions are relatively evenly distributed across the gene. PD is a natural lipoprotein whose signal sequence contains a consensus sequence for bacterial lipoproteins, Leu-Ala-Gly-Cys, as amino acids 16 to 19[58]. There are a minimum of 12 lipoproteins in *H. influenzae*[59], one of which is PD. When the lipoprotein is secreted, the amino-terminal cysteine residue is modified after translation through added glycerol moiety containing ester-linked fatty acids. In the next step, the signal peptide is cleaved by signal peptidase II specific for lipoproteins, and the newly-formed amino terminus is further acylated with an amide-linked fatty acid. Therefore, membrane anchor is developed by the fatty acids linked to the amino-terminal cysteine residue[60].

No specific functions were assigned to PD; however, *in vivo* and *in vitro* studies have shown that PD has a role in NTHi pathogenesis due to the absence of antigenic drift and surface localization. Despite the fact that it is not an adhesion PD can indirectly stimulate attachment and invasion via the glycerophosphodiester phosphodiesterase activity required for transferring choline from the host to LOS of *H. influenzae*[60,61]. Bacterial invasion is enhanced by signaling ChoP+LOS variants through the platelet- activating factor receptor[62,63]. Glycero-phosphorylcholine is one of the greatest degradation products of eukaryotic membrane-associated phosphor-lipids that can be hydrolyzed by PD because of its GlpQ activity. Glycerophosphorylcholine has the ability to produce glycerol 3-phosphate and choline in order to achieve choline directly from host epithelial cells[64]. The phosphorylcholine-decorated LOS serves as a ligand for the platelet-activating factor receptor of bronchial epithelial cells. In a chinchilla model of AOM, the presence of phosphorylcholine leads to the increased formation of stable biofilm communities of NTHi[65].

PD has shown 67% structural identity with *glpQ* encoded by the periplasmic glycerophosphodiester phosphodiesterase enzyme in *Escherichia coli*. This periplasmic glycerophosphodiesterase, encoded by *glpQ*[66,67], is a membrane-bound protein in *H. influenzae* through fatty acids linked to a cysteine residue in a consensus sequence for bacterial lipoproteins[68,69]. PD, mutated with no cysteine residue, is hydrophilic and could not be acylated. The nonacylated PD can be secreted into the periplasmic space of *E*. *coli*, indicating the role of acylation in hydrophobicity of PD as an outer membrane-anchored lipoprotein[68]. PD also increases bacterial adhesion and internalization into human monocytes. *In vivo* and *in vitro* studies have demonstrated that the NTHi virulence in the upper respiratory tract is related to PD expression. For instance, a 100 fold decrease in virulence was found in a PD-deficient strain compared to the PD-expressing strain in a rat model of otitis media. In addition, culture specimens, inoculated with the PD-deficient NTHi compared with isogenic wild type NTHi, indicated a reduction in the onset of ciliary activity after 12 hours. Ciliary dysfunction was significantly higher in the PD-expressing strain, as compared to the PD-negative mutant (*P*<0.01). In addition, a significant loss of cilia was observed in the PD-expressing strain after 48-hour incubation, indicating that PD is responsible for etiology of upper respiratory tract infection caused by NTHi, probably due to increased functional and morphological damages to ciliated epithelial cells. Based on a study on animal models, the protective antibodies against NTHi otitis media in rat and chinchilla were provoked by PD[42,69-72].

#### D15 protein

OMP D15, a protein with a molecular weight of approximately 80 kDa, is available in both encapsulated and nonencapsulated *Hemophilus* strains[73].

#### HMW1 and HMW2 (high molecular weight 1 and 2) proteins

HMW proteins, such as HMW1 and HMW2, are found in about 70% of *Hemophilus* strains. These proteins have heterogeneity area in different nontypeable *Haemophilus* strain. The remaining 25% of *Hemophilus* strains lack the protein, which is an immunogenic protein expressed in *Hemophilus influenzae* type a (Hia)[63].

#### Hia protein

Hia is one of the most important binding proteins that is expressed in some strains of Hib and provides an efficient attachment to the epithelial cells. Hia is a surface-exposed protein whose amino acids 221-779 are involved in the binding. The protein is a member of the autotransporter family, and almost all of the strains have this protein, except for the strains having lost HMW proteins[74].

#### Hap (heterogeneous nuclear ribonucleoprotein A1-interacting protein)

Hap is a binding protein found in all or most strains of *Hemophilus*, showing a high level of protection in many animal model studies. The protein is a member of the autotransporter family that allows bacteria to attach to epithelial cells and extracellular matrix proteins, facilitating the possibility of bacterial aggregation and biofilm formation[75,76].

## CONCLUSION

NTHi is one of the most important causes of acute middle ear infections in children and respiratory diseases in adults. Since the introduction of conjugate vaccines against the bacteria encapsulated Hib in the late 1980s, the prevalence of invasive diseases caused by Hib has decreased significantly in developed countries; however, invasive diseases caused by NTHi strains have spread and, in many areas, lead to invasive diseases. The lack of a protection capsule, high antigenic heterogeneity, and high changes in exposed antigens are significant barriers to the development of effective vaccines against NTHi. Therefore, studies investigating vaccine development have focused on the protectively area of the external membrane proteins, LOS, and pili. To this end, various vaccine candidates have been developed with the immunogenicity of PD, providing high protection against NTHi.
